# Intensified vmPFC surveillance over PTSS under perturbed microRNA-608/AChE interaction

**DOI:** 10.1038/tp.2016.70

**Published:** 2016-05-03

**Authors:** T Lin, A Simchovitz, S Shenhar-Tsarfaty, S Vaisvaser, R Admon, G Hanin, M Hanan, E Kliper, Y Bar-Haim, N Shomron, G Fernandez, G Lubin, E Fruchter, T Hendler, H Soreq

**Affiliations:** 1 The Tel-Aviv Center for Brain Function, Wohl Institute for Advanced Imaging, Tel Aviv Sourasky Medical Center, Tel Aviv, Israel; 2 School of Psychological Sciences, Tel Aviv University, Tel Aviv, Israel; 3 The Edmond and Lily Safra Center for Brain Science, The Silberman Institute of Life Sciences, The Hebrew University of Jerusalem, Jerusalem, Israel; 4 Sagol School of Neuroscience, Tel Aviv University, Tel Aviv, Israel; 5 Department of Cell and Developmental Biology, Sackler Faculty of Medicine, Tel Aviv University, Tel Aviv, Israel; 6 Donders Institute for Brain, Cognition and Behaviour, Department for Cognitive Neuroscience, Radboud University Medical Centre, Nijmegen, The Netherlands; 7 Division of Mental Health, Medical Corps, Israel Defense Forces, Israel; 8 Department of Physiology and Pharmacology, Sackler Faculty of Medicine, Tel Aviv University, Tel Aviv, Israel

## Abstract

Trauma causes variable risk of posttraumatic stress symptoms (PTSS) owing to yet-unknown genome–neuronal interactions. Here, we report co-intensified amygdala and ventromedial prefrontal cortex (vmPFC) emotional responses that may overcome PTSS in individuals with the single-nucleotide polymorphism (SNP) *rs17228616* in the acetylcholinesterase (AChE) gene. We have recently shown that in individuals with the minor *rs17228616* allele, this SNP interrupts AChE suppression by microRNA (miRNA)-608, leading to cortical elevation of brain AChE and reduced cortisol and the miRNA-608 target GABAergic modulator CDC42, all stress-associated. To examine whether this SNP has effects on PTSS and threat-related brain circuits, we exposed 76 healthy Israel Defense Forces soldiers who experienced chronic military stress to a functional magnetic resonance imaging task of emotional and neutral visual stimuli. Minor allele individuals predictably reacted to emotional stimuli by hyperactivated amygdala, a hallmark of PTSS and a predisposing factor of posttraumatic stress disorder (PTSD). Despite this, minor allele individuals showed no difference in PTSS levels. Mediation analyses indicated that the potentiated amygdala reactivity in minor allele soldiers promoted enhanced vmPFC recruitment that was associated with their limited PTSS. Furthermore, we found interrelated expression levels of several miRNA-608 targets including CD44, CDC42 and interleukin 6 in human amygdala samples (*N*=7). Our findings suggest that miRNA-608/AChE interaction is involved in the threat circuitry and PTSS and support a model where greater vmPFC regulatory activity compensates for amygdala hyperactivation in minor allele individuals to neutralize their PTSS susceptibility.

## Introduction

Posttraumatic stress disorder (PTSD) is a severely debilitating psychiatric condition, especially in combat soldiers at high risk for trauma or chronic stress exposure.^1, 2^ PTSD, however, develops only in a subset of those experiencing such events,^3^ pointing to individual differences in vulnerability and resiliency. The variable responses to stressful situations manifest in healthy stress-exposed individuals as subthreshold posttraumatic stress symptoms (PTSS), known to increase the risk for PTSD^4^ and cause impairments in daily life situation,^5, 6^ including difficulties in emotion regulation.^7^ Although several neural and genetic risk factors for PTSS are known,^8, 9, 10, 11^ the molecular mechanisms underlying the variable intensity of such symptoms remain elusive.

Most imaging genetic studies in PTSS and PTSD focused on polymorphisms in coding regions of candidate genes.^9, 12, 13^ However, the great majority of genetic polymorphisms are located in noncoding regulatory regions.^14^ One of these is the single-nucleotide polymorphism (SNP *rs17228616*) C2098A substitution in the ‘seed' domain of miRNA-608 binding site, at the 3′-untranslated region of the acetylcholinesterase (AChE) gene. We have recently shown that individuals with the minor A-allele demonstrate weakened interaction between miRNA-608 and the AChE gene.^15^ These individuals also exhibit reduced cortisol and their cortical tissues present excessive brain AChE hydrolytic activity, accompanied by decreases in other predicted neuronal protein targets of miRNA-608, including the Rho GTPase CDC42 involved in GABAergic signaling and the inflammation-regulating cytokine interleukin 6 (IL-6). These effects were attributed to weakened AChE interaction with the primate-specific regulatory miRNA-608,^15, 16^ and are all stress-associated and predicting PTSS and PTSD.^15, 17, 18^ Intriguingly, a recent genome-wide association study has reported significant association between the rs78011900 SNP, which is in full-linkage disequilibrium with *rs17228616*, and hypertension^19^ that is also related to PTSD.^20^ As the predicted cholinergic/GABAergic and inflammatory imbalance may modify the inherited risk or resiliency to develop stress-related symptoms following stressful challenges, we initiated a study of the functional consequences of this genetic variation on the development of PTSS symptoms that indicate a risk of PTSD and the relation of the rs78011900 SNP to the threat neural circuitry.

The amygdala, hippocampus and prefrontal cortex have interrelated roles in PTSS and PTSD pathophysiology^8, 21, 22^ and receive high levels of cholinergic and miRNA inputs.^23, 24^ Rodent studies show that pharmacological and molecularly induced suppression of hippocampal AChE activity associate with increased anxiety- and depression-like behaviors and decreased resilience to repeated stress.^25^ This plausibly involves stress-impaired hippocampal inhibitory feedback to the amygdala,^26^ which may lead to enhanced amygdala reactivity, a hallmark and a predisposing factor of PTSD.^8, 24, 27, 28, 29^ Excessive acetylcholine (ACh) release in the amygdala strengthens the associations between environmental stimuli and stressful events, potentially contributing to maladaptive learning and underlying affective disorders.^30^ Human studies indeed demonstrate that increases in ACh signaling contribute to stress-related illnesses, such as major depression.^31^ As the *rs17228616* polymorphism is related to excessive brain AChE hydrolytic activity,^15^ we surmised that the AChE gene is close to its maximal expression capacity in individuals with the minor allele. This would limit their ability to react to a changing environment by overproducing AChE to suppress the induced stress. We expected that this would be manifested by enhanced neural response of the threat circuit, including the amygdala which is rich in both GABAergic synapses and AChE.^32^ In addition, animal studies have demonstrated involvement of inherited tendencies of AChE increases in response to stress,^33^ miRNA suppression of brain AChE (by miRNA-132) which limits stress-induced cognitive impairments,^34^ and CDC42 suppression leading to anxiogenic reaction.^15^ Therefore, we predicted modulated PTSS reactions in individuals with the minor allele of the *rs17228616* SNP.

How individuals compensate for risk-associated genotypes is a challenging, scarcely studied issue. A neural candidate for coping with exaggerated amygdala activity is the ventromedial prefrontal cortex (vmPFC). This region is activated when a negative emotional response is suppressed, in a manner related to the elimination of previously learned associations of threat-conditioned signals.^35, 36^ Under normal conditions, suppressing messages by the vmPFC lead to amygdala downregulation in response to negative stimuli, whereas PTSD patients show less activity in the vmPFC than healthy volunteers when experiencing or regulating emotions.^37^ This indicates vmPFC involvement in the process of regulation and thus resilience to trauma. In the current study, we used functional magnetic resonance imaging (fMRI) to examine how the *rs17228616* polymorphism in the AChE gene impacts PTSS following chronic military stress exposure; specifically, we asked whether it serves as a vulnerability, resilience or an inert factor, and what are its functional influences on the human amygdala and vmPFC responses to stressful challenges.

## Materials and methods

### fMRI experiment

#### Participants

The participants were 76 Israel Defense Forces soldiers (13 females; age 18–19 years) who experienced 1 year of chronic military stress during their mandatory service. Of those, 29 participants (Group A) served as combat paramedics and 47 (Group B) were recruited after 1 year of intensive and advanced combat training. During military training, the participants were exposed to a wide variety of stressful physical and psychological demands, including sleep restrictions, prolonged periods of physical survival challenges, face-to-face combat training and counter-terrorism combat course, that have been shown to affect well-being^38^ and may impact the development of stress-related symptoms.^1, 2^ The fMRI data of Group A was already published by our group.^29^ As there were no differences in amygdala and vmPFC activity or in PTSS between paramedics and soldiers in training (all F<1.28, *P*>0.25), all 76 volunteers were considered as a single group. All participants had no reported history of psychiatric or neurological disorders, no current use of psychoactive drugs, no family history of major psychiatric disorders and no incidence of childhood abuse or potentially traumatic events before military enrollment.

#### Genotyping

**Figure 1:**
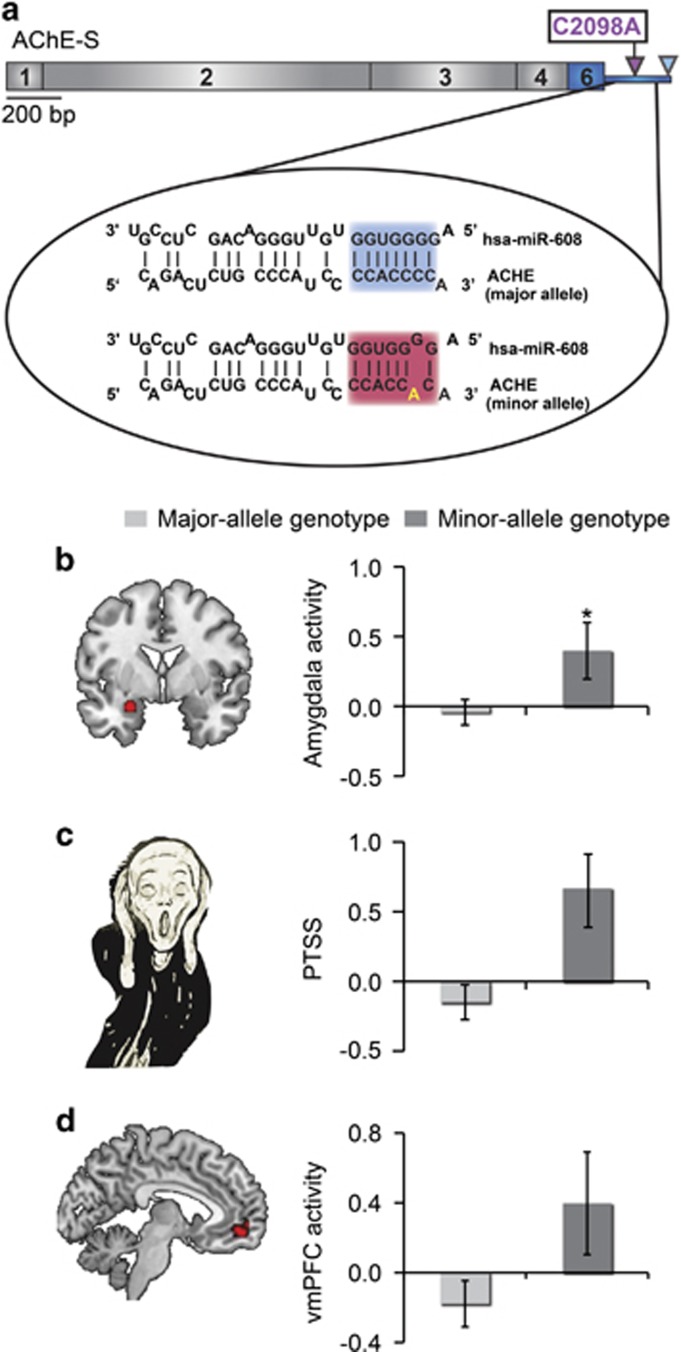
*rs17228616* single-nucleotide polymorphism impairs hsa-miR-608/AChE interaction. (**a**) Schematic illustration of the AChE transcript, exon numbers and the *rs17228616* location, with the C to A (C2098A) major to minor allele substitution interrupting hsa-miR-608 binding. (**b**) Increased activation within the amygdala region of interest (ROI; −28, 0, −21) for the minor allele (AA, AC, *n*=13) relative to the major allele (CC, *n*=63) group. (**c**) Limited elevation in posttraumatic stress symptoms (PTSS, *z*-score) in the minor allele (AA, AC, *n*=13) relative to major allele (CC, *n*=58) group. (**d**) Increased activation within the ventromedial prefrontal cortex (vmPFC) ROI (0, 50, −12) for the minor allele (AA, AC, *n*=13) relative to the major allele (CC, *n*=63) group. The data are represented as mean±s.e. Bar charts in **a** and **c** show the mean of beta values across all voxels in the ROIs for emotional versus neutral stimuli. *P<0.05. AChE, acetylcholinesterase.

DNA was extracted from saliva samples collected with an OrageneTM DNA Self-Collection Kit in Group A and from blood samples in group B, using commercial kits. Genotyping of the A-allele of *rs17228616* (C2098A) versus the C-allele (Figure 1a) was performed using TaqMan genotyping primers and AccuStart genotyping ToughMix low ROX (Quanta BioSciences, Gaithersburg, MD, USA). To further differentiate between homozygous (AA) and heterozygous (CA) *rs17228616* individuals, PCR-amplified DNA was fully sequenced. The participants were categorized according to DNA sequencing: carriers of one or two copies of the minor A-allele of *rs17228616*C (AA/CA) were assigned to the minor allele group (*N*=13), whereas C homozygotes (CC) were assigned to the major allele group (*N*=63).

#### fMRI visual task

The participants viewed backward-masked colored photographed pictures of three contents (military, medical and neutral) presented for either 33 or 83 ms in a block design and were asked to indicate whether they recognized a person or an object as described in ref. 29 (see Supplementary Materials and Methods for details).

#### Assessment of PTSS

The PTSS levels in Group A were evaluated using the posttraumatic stress diagnostic scale questionnaire (PDS).^39^ In Group B, the symptoms were evaluated with the military version of the 17-item PTSD checklist,^40^ which specifically asks about symptoms related to stressful military experiences. As each of these questionnaires uses different scales, we calculated *z*-scores for each group, enabling to integrate data from both groups. Five participants did not complete the PTSS questionnaires, and thus were excluded from analyses concerning this variable.

#### Mediation analysis

The mediation indirect effects were tested using the PROCESS macro in SPSS,^41^ which tests indirect effects using a bootstrap approach. The indirect effect was considered to be significant if its 95% bootstrap confidence intervals from 10 000 iterations did not include zero at *P*=0.05. In all indirect effect analyses, A-allele carriers were coded as 1.

### Amygdala brain samples

The dissected amygdala tissues from non-demented control volunteers (*n*=7, three males, four females) were provided by the Netherlands Brain Bank (Supplementary Table S1). Written informed consent from donors or their next of kin, as well as ethical approval was obtained in all the cases. The tissues were kept at −70 degrees until RNA extraction, and were used for quantitative PCR analysis. RNA extraction from brain tissues, cDNA synthesis, quantitative PCR measurements and data analysis were essentially performed as in refs 15, 16 (see Supplementary Materials and Methods for details). DNA was extracted as described in ref. 15 followed by genotyping of PCR product amplification via Illumina sequencing. Unfortunately, we could not find human vmFPC tissues, so we could not extend these tests to the vmPFC.

## Results

The coding regions of the genome account for less than 2% of the entire human DNA,^42^ and numerous noncoding genomic domains contribute to individual differences in reactivity to neural diseases, including threat and its regulation.^43, 44^ Nevertheless, most anxiety-related imaging genetic studies focus on those simple-to-explain polymorphisms in the coding regions of candidate genes.^9^ In the present study, we selected the *rs17228616* SNP in the noncoding region of the AChE gene to investigate gene–environment brain interactions as those are reflected in PTSS. This was achieved by combining genotype and brain transcript tests with fMRI measurements of amygdala and vmPFC responses to emotional stimuli in a military population at high risk for chronic stress exposure, known to impact the development of stress-related symptoms.^1, 29^

### Genotyping results

The *rs17228616* SNP in the AChE gene was present in 17% of 76 participants, fourfold higher incidence than in Caucasians but twofold less than in African American.^15^ Thirteen participants (17.1%) formed the minor A-allele group (AA, AC) and 63 participants (82.9%) with the CC genotype formed the major allele group (Figure 1a). This distribution accords with another study from our group identifying the minor allele variant in 17% of over 900 tested Israeli participants (Shenhar Tsarfaty *et al.*, in preparation). The ethnic differences in the prevalence of this SNP are compatible with evolution studies of miRNAs in population migrations^45^ that should be considered in functional studies of the brain-related implications of this and other polymorphisms.

### Genotype effects on limbic responses and PTSS following emotional stimuli

The amygdala and hippocampus are rich in both GABAergic synapses^46^ and AChE,^32, 47^ suggesting that their activity may be modulated by the cholinergic/GABAergic imbalance imposed by the *rs17228616* polymorphism in the AChE gene. Compatible with this prediction, genotypes with the minor allele (AC, AA) were associated with greater amygdala reactivity to emotional stimuli compared with other volunteers (Mann–Whitney *U*=269.0, *P*<0.05; Figure 1b). In contrast, the *rs17228616* SNP did not influence hippocampus function (Mann–Whitney *U*=308.0, *P*=0.16). Moreover, despite their neurogenetic vulnerability,^8, 15^ PTSS levels did not differ between minor allele and major allele individuals (Mann–Whitney *U*=267.0, *P*=0.09; Figure 1c). This accords with recent genome-wide association studies, concluding that the genetic architecture of PTSD may be determined by many SNPs with small effects that are not directly related to the cholinergic pathway.^48, 49^ For the AChE gene, this outcome plausibly reflects the exceedingly high G, C content that complicates genome-wide association study data collection. It is also possible that, in this study, the small sample size and low variability in PTSS hindered the detection of a genotype–PTSS association.

### Genotype effects on prefrontal responses to emotional stimuli

The great majority of stressed individuals cope with their inherited genome features and do not develop excessive PTSS or PTSD. To explore a possible route that enables minor allele individuals to compensate for the imbalanced amygdala signaling, we compared the reactions of their vmPFC, a key component of the cortico-limbic circuit involved in the top-down regulation of affective subcortical circuitry, to those of their major allele peers. The *rs17228616* minor allele group members showed marginally significant threefold higher vmPFC activation than the major allele group to emotional versus neutral stimuli (Mann–Whitney *U*=272.0, *P*=0.058; Figure 1d). Although this finding is only marginally significant, we speculated that it might indicate that greater vmPFC functioning may operate as a clinically helpful compensatory mechanism for A-allele individuals to neutralize their sensitivity to stress, and that they present no increase in PTSS owing to the recruitment of the vmPFC to overcome their stress vulnerability.

### Relationship between genotype, brain response and PTSS

**Figure 2:**
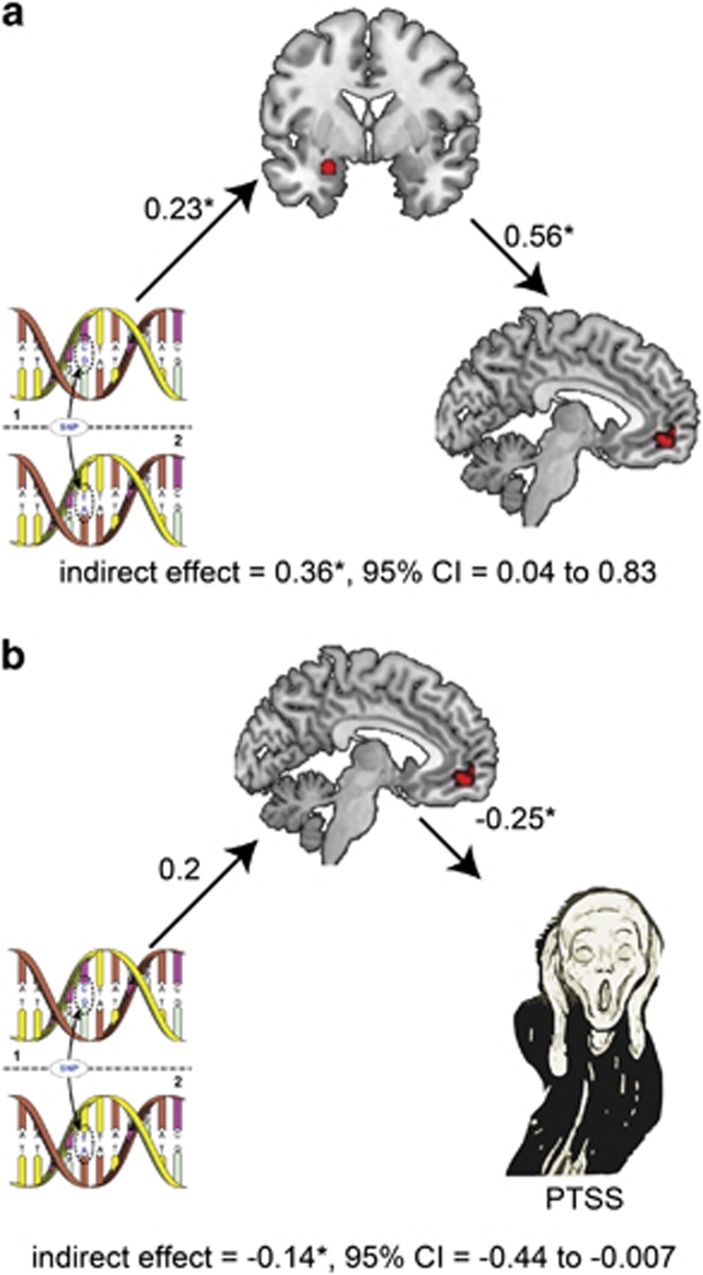
Models of the relationship between *rs17228616* genotype, brain activity and posttraumatic stress symptom (PTSS). (**a**) The illustrated mediation model depicts a significant indirect path of the effect of genotype on ventromedial prefrontal cortex (vmPFC) activity via amygdala activation. Specifically, A-allele carriers exhibited greater amygdala reactivity to emotional stimuli, which in turn was associated with increased vmPFC activity. (**b**) The illustrated mediation model depicts a significant indirect effect of genotype on PTSS via vmPFC activation. Specifically, A-allele carriers exhibited greater vmPFC response to emotional stimuli, which in turn was associated with lower levels of PTSS symptoms. The β-values are shown next to the arrows indicating each link in the analysis. *P<0.05. 95% CI, 95% confidence interval.

To test the above-mentioned theory in the search for potential functional relevance of the neural patterns that might explain the limited difference in PTSS between minor and major allele individuals, we conducted an indirect effect analysis using a bootstrapping approach.^50^ This method avoids the power problem introduced by non-normality and is less restricted by sample size. This mediation analysis revealed that volunteers with the minor allele exhibited exacerbated amygdala reactivity to emotional stimuli, which in turn was associated with increased vmPFC activity (indirect effect=0.36*, 95% confidence interval: 0.04 to 0.83; Figure 2a). The observed pattern of vmPFC hyperactivity accompanied by increased amygdala activity as found in the minor allele group is strikingly different from previous reports of hyperresponsive amygdala accompanied by hyporesponsive vmPFC in PTSD,^51^ suggesting that minor allele individuals may possibly achieve limited PTSS by over-recruiting the vmPFC. To verify this conclusion, a second mediation model was tested, which yielded a significant indirect effect of genotype on PTSD symptoms via vmPFC activation (indirect effect=−0.14*, 95% confidence interval: −0.44 to −0.007; Figure 2b), such that the greater vmPFC response to emotional stimuli in volunteers with the A-allele was associated with lower levels of PTSS compared with major allele carriers. Conversely, the indirect effect of genotype through the amygdala was insignificant (indirect effect=−0.09, 95% confidence interval: −0.39 to 0.03), compatible with the minor allele group's limited increase of trait anxiety^15^ and PTSD symptoms (Figure 1b). Although mediation analyses should be interpreted with caution and replications of these results are required to draw firm conclusions, we conclude that the *rs17228616* polymorphism exacerbates amygdala activity, but is not a PTSS risk factor as it promotes vmPFC hyperactivation, which overcomes the exaggerated amygdala response.

### Expression of the primate-specific miRNA-608 and its targets in the human amygdala

**Figure 3:**
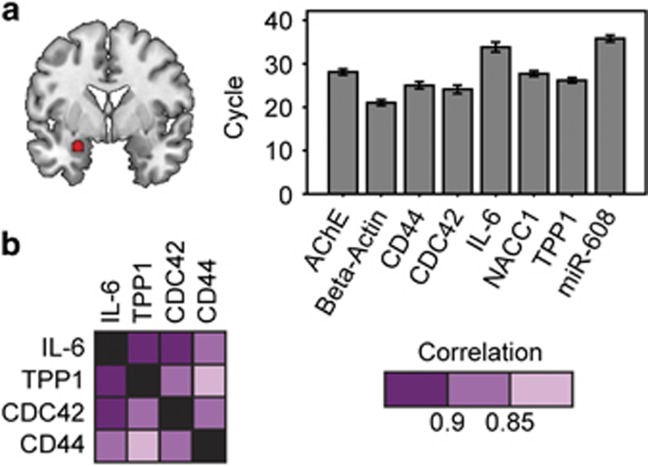
Interrelated expression of miR-608 and its targets in the amygdala. (**a**) Human amygdala (marked red) served to extract RNA. hsa-miR-608, as well as six of its verified and predicted targets, including acetylcholinesterase (AChE) and CDC42 and the normalizing beta-actin gene are all expressed in the human amygdala (quantitative PCR (qPCR) cycle <34 for targets, qPCR cycle <36 for miRNA-608). Shown are Cq values as measured by qPCR. (**b**) Several pairs of verified and predicted targets of hsa-miR-608 show positively correlated expression patterns, normalized to beta-actin and RIN (RNA integrity number) values (*P*<0.05, false discovery rate corrected). Color code shows correlation matrix intensity. IL-6, interleukin 6; miRNA, micro RNA.

Animal studies demonstrate that miRNAs expressed in the amygdala may be involved in the stress response,^52^ but previous tests of miRNA-608 and its targets in the human brain were limited to the temporal cortex.^15^ To assess expression of miRNA-608 and its target genes in the amygdala, we performed quantitative PCR with reverse transcription measurements from amygdala tissues of healthy human adults received from the Netherlands Brain Bank. These analyses confirmed expression, albeit at a modest level of miRNA-608 in the amygdala of seven donors, all homozygotes for the major allele of the *rs17228616* SNP (Figure 3a). We also observed expression of several miRNA-608 targets, including AChE in these tissues (Figure 3a). Moreover, our measurements revealed positive correlations between several pairs of both predicted and verified brain-expressed miRNA-608 target transcripts, following RNA quality and beta-actin normalization. For the seven brains, we observed significant positive correlations between the GABAergic regulating CDC42, the immune biomarker CD44, the pro-inflammatory cytokine IL-6 and the telomere-related TPP1 transcript (all correlations passed false discovery rate correction of 0.05, see Table 1 and Figure 3b). The fact that these transcripts are all expressed in the amygdala is essential for and compatible with the proposed mechanism of action; and the correlated expression levels indicated common regulatory mechanism(s) for these transcripts in the human amygdala, which could include miRNA-608-mediated control. These findings further predicted that higher brain AChE levels, as shown for individuals with the minor allele, would be associated with reduced and interrelated levels of CDC42, CD44 and IL-6. This can imbalance cholinergic and GABAergic signaling, and promote inflammation and consequent anxiety^15^ (Figure 3b). There was no apparent effect of age or gender.

## Discussion

**Figure 4:**
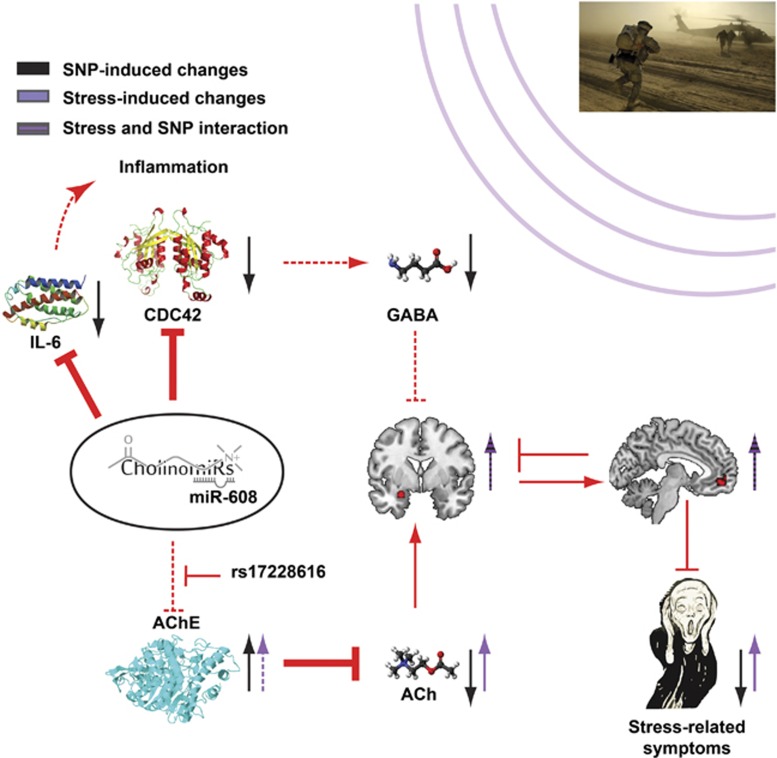
Schematic presentation of how the *rs17228616* single-nucleotide polymorphism (SNP) in the acetylcholinesterase (AChE) gene may affect the vulnerability to stress. This SNP interrupts miRNA-608/AChE interaction, leading to (1) elevated brain AChE activity and thus reduced cholinergic signaling, (2) reduction in the GABAergic modulator CDC42, limiting the inhibitory GABAergic signaling and (3) reduced IL-6 levels, effecting inflammatory pathways. Together, these signaling pathway impairments may enhance amygdala reactivity to emotional stimuli of minor allele individuals due to insufficient suppression of stress-related increases in cholinergic signaling and reduced inhibitory GABAergic signaling. As a coping mechanism under trauma load, these individuals over-recruit the ventromedial prefrontal cortex to downregulate the amygdala, and thus develop limited posttraumatic stress syndrome. Broken arrows represent reduced inhibitory input. The effects of this SNP, of stress exposure and of SNP-exposure interactions are represented by black, purple and striped black/purple arrows, respectively. IL-6, interleukin 6; miRNA, micro RNA.

The *rs17228616* SNP interferes with AChE suppression by the cholinergic controller and primate-specific miRNA-608 (‘CholinomiR',^53^), and its occurrence in healthy adults is associated with multiple anxiety- and inflammation-related changes: elevated brain AChE activity and correspondingly reduced brain levels of the GABAergic-related Rho GTPase CDC42, suppression of which induces anxiety, and the inflammation-regulating cytokine IL-6.^15^ MiRNA-608 has recently been identified as a tumor-suppressive miRNA,^54, 55^ and has been proposed as a potential novel therapeutic target for treating osteoarthritis.^56^ Yet, because miRNA-608 is primate-specific,^45^ previous animal experiments of miRNA/AChE interactions and stress^34^ cannot be reproduced here to determine a causal link to stress. Despite this difficulty, the current study points to the involvement of miRNA-608 in stress-related neural reactions. We demonstrated that following chronic stress, individuals with the *rs17228616* minor allele enhance neural activity in the amygdala accompanied by non-pathological PTSS levels. This finding was supplemented with a marginal increase in vmPFC response. Our model predicts multileveled effects of this polymorphism on the neural response to emotional stimuli within the threat circuit and its association with PTSS, as is schematically summarized in Figure 4. We further found expression of miRNA-608 and several of its verified and predicted target transcripts in the amygdala, including AChE, CDC42, CD44, NACC1 and IL-6; A subset of these, involved in inflammation, showed very tight positive interrelated correlations (CDC42, CD44, IL-6 and TPP1), indicating common regulation by miR-608, among other potential causes. Our previous work has shown such correlation in the temporal gyrus, expanding the implications of our current analyses beyond the amygdala. To the best of our knowledge, our current study is the first to implicate a SNP in a noncoding region in measurable brain fMRI changes under emotional stimuli, and the first to suggest a functional coping mechanism with a risk-associated SNP.

Although PTSS were not directly related to the polymorphism, our fMRI results provide support for the relation between the *rs17228616* polymorphism and PTSS by demonstrating that individuals with the *rs17228616* minor allele show heightened amygdala response to emotional stimuli. This is compatible with earlier functional neuroimaging studies reporting exaggerated amygdala activation in patients with PTSD compared with control subjects^27, 28^ and support the notion that amygdala hyperactivation is a predisposing factor for developing stress-related disorders.^8, 29^ The finding that minor allele individuals show hyperactive amygdala but do not present elevated PTSS has led us to jointly investigate how they cope with stressful challenges.

The *rs17228616* minor allele group showed a trend of increased vmPFC activation compared with the major allele group. This finding was further strengthened by a significant indirect path analysis showing that soldiers with the minor A-allele also exhibited higher levels of amygdala activity compared with those with the major C-allele, and that the minor allele was involved in increased recruitment of the vmPFC in these volunteers. The results of this mediation analysis suggest that minor allele individuals may recruit the vmPFC to downregulate their elevated amygdala reactivity more effectively than the major allele group. An additional mediation analysis suggested that the elevated vmPFC activity has a clinically protective role in A-allele volunteers, such that they attained lower than predicted levels of PTSS compared to major allele carriers by increasing the recruitment of their vmPFC. However, other, yet-unknown explanations for these fMRI results may exist. To validate or exclude this hypothesis, future studies could test whether parallel vmPFC hyperactivation occurs in other cases where the inherited tendency to develop PTSS is compensated for, and whether PTSD patients with the A-allele lack this compensatory mechanism and thus develop and maintain high levels of symptoms.

Amygdala enhancement in minor allele individuals may be driven by either the imbalanced cholinergic, GABAergic and/or inflammation signaling, which are all intensified under stressful environments^57^ (Figure 4). Apart from their direct effects on the amygdala, GABA and acetylcholine could possibly influence this phenotype indirectly through other regions within the threat circuit, such as the hippocampus.^24, 58, 59^ However, the *rs17228616* SNP was not associated with modified hippocampus activity, suggesting that individual differences in the AChE system likely explain only part of the neural changes associated with PTSS. This finding also excludes hippocampal involvement in the limited PTSS of minor allele individuals. An alternative explanation is that although their reduced cholinergic signaling causes decreased hippocampal activity, the hippocampus is upregulated back to normal levels by the overrecruitment of the vmPFC.^35^ To elucidate this, focused studies of the effects of this gene on hippocampus–vmPFC connectivity are needed.

The noncoding SNPs affecting higher brain functions and chronic disease include a polymorphism in the 3**′**-untranslated region of the brain-expressed human Slit and Trk-like 1 (*SLITRK1*) gene, which strengthens an existing miR-189 target site, supports neurite growth and is involved in Tourette's syndrome and attention-deficit hyperactivity disorder.^60^ Also, college students homozygous for the ancestral A variant of an A>G polymorphism, which changes miRNA-96 regulation of the aggressive behavior-associated *HTR1B* gene, exhibit more conduct-disorder behaviors than individuals with the G allele.^61^ Our findings, in comparison implicate the ability to cope with miRNA dysregulation in brain-related diseases. The genetic variations evident in the miRNA-608 gene itself (the 'seed' region) may alter its binding and thus activity.^62^ Consequently, in addition to the 3′-untranslated region polymorphism, miRNA polymorphisms can potentially serve as additional biomarkers for PTSS, which warrants investigation.

There are several limitations to this study. The neurocircuitry models posit that in PTSD, the vmPFC fails to inhibit the amygdala, which leads to exaggerated threat responses and emotion regulation deficits.^37^ The currently observed effect thus reflects differential increases in the reactivity of the amygdala following prolonged exposure to stress and trauma.^63^ However, the genotype effect may also reflect pre-trauma vulnerability factors rather than consequences of stressful situations.^8, 29^ The prospective studies examining the relation between the *rs17228616* polymorphism and amygdala activity before and after stress insults of variable impact and length are required to disentangle this question. In addition, the currently studied population consisted of healthy Israel Defense Forces soldiers selected by extensive mental and physical screenings, and the *rs17228616* polymorphism may have a different effect in the general population; genuine highly anxious populations should be tested before the results can be generalized.^64^ However, the use of a relatively healthy sample minimizes confounding factors such as comorbidities and medication use, thus representing a good approach for studying PTSS as a continuum. Nevertheless, the use of a small homogenous sample may have reduced PTSS variability, weakening the statistical power to detect all statistical effects, thus warranting additional studies on larger and more variable trauma-prone populations, such as paramedics or firefighters. Further, the sample size of amygdala tissues and soldiers participating in the fMRI experiment may be too small to detect all the associations. It is also possible that the results of this study may be only applicable to chronic cases and not to acute stress symptoms.

To conclude, using a unique population at high risk for chronic stress exposure, we found that the *rs17228616* gene variant influences neural responses to emotional stimuli; but that in the context of trauma load, its effect on PTSS is limited, plausibly due to intensified recruitment of the vmPFC. Importantly, our findings support the concept that noncoding RNAs may be involved in PTSS, and should be integrated into a neural model of the disorder. This study highlights the effectiveness of combined genomic and neuroimaging methods in the identification of intermediate phenotypes for PTSS and other psychiatric disorders and provides a further step towards personalized medicine in psychiatry by identifying previously neglected genetic variants of disease risks that may contribute to its vulnerability. Notably, our current findings point to one possible PTSS risk pathway, in which a risk genotype predisposes individuals to increased fear reactivity. This involves exaggerated amygdala responses that are supposedly downregulated by the vmPFC to avoid the development of PTSS. These findings also point to potential new treatment avenues by neuromodulation techniques targeting the amygdala or amygdala–vmPFC interactions.

## Figures and Tables

**Table 1:** Correlations between brain-expressed miRNA-608 target transcripts

	*CD44*	*CDC42*	*IL-6*	*TPP1*
CD44	1	0.901 (0.028)	0.983 (0.002)	0.900 (0.028)
CDC42	0.901 (0.028)	1	0.861 (0.033)	0.849 (0.033)
IL-6	0.983 (0.002)	0.861 (0.033)	1	0.865 (0.033)
TPP1	0.900 (0.028)	0.849 (0.033)	0.865 (0.033)	1

Abbreviations: IL-6, interleukin 6; miRNA, micro RNA.

Significant correlations between pairs of transcripts are shown along with the respective post-false discovery rate *P*-values (in parentheses).
