# Supplementary material for: Intensified vmPFC surveillance over PTSS under perturbed microRNA-608/AChE interaction
Source: Transl Psychiatry. 2016 May 3;6(5):e801–. doi: 10.1038/tp.2016.70 (PMC5070052; doi:10.1038/tp.2016.70)
Supplement: Supplementary Information [file tp201670x1.docx]

**Supplementary Material and Methods**

***fMRI experiment***

*fMRI visual task*. Visual stimuli were presented in a parametric block design fashion. Participants viewed colored photographed pictures of 3 contents (military, medical, and neutral), half of which were people and half objects. Each picture was presented for 33 or 83 ms immediately followed by a scrambled image for 477 or 427 ms, respectively (i.e., backward masking). All images were shown only once during a scan. The inter-stimulus interval was 500 ms, during which a blank gray background was presented. The paradigm was composed of 28 epochs (each 9 sec long) and consisted of a single content interleaved with 6- or 9-sec margins of a blank gray screen. A total of 216 pictures were presented at a rate of 1 Hz. Two versions of the paradigm were counterbalanced between participants to control for the effects of order. One mixed-content epoch and a blank epoch of 12 sec were shown at the beginning of the experiment for practice and were not included in the analysis. To control for equal attention throughout the paradigm, the participants were asked to indicate whether they recognized a person or an object in the picture by pressing a key after each picture.

*Brain imaging acquisition and analysis*. Brain scanning was performed on a GE 3T SignaHDxt MRI scanner with an eight channels head coil. Functional images were acquired using a single-shot echo-planar T2*-weighted sequence. The following parameters were used: TR/TE: 3000/35; flip angle 90; FOV: 20X20 cm1; matrix size: 64X64; 44 axial slices with 3 mm thickness and no gap covering the entire brain. Acquisition orientation was of the fourth ventricle plane. In addition, each functional scan was accompanied by a three-dimensional scan using T1-SPGR sequence (1×1×1 mm^3^). Data were preprocessed and analyzed using conventional statistical parametric mapping (SPM5). fMRI data preprocessing included correction for head movement (subjects with movement above 2 mm were discarded), realignment, normalizing the images to Montreal Neurological Institute (MNI) space and spatially smoothing the data (FWHM, 6 mm). The first six functional volumes, before signal stabilization, were excluded from the analysis.

Statistical maps were prepared for each participant using a general linear model (GLM), in which the six task conditions were defined as district predictors (medical content 33 ms, medical content 83 ms, army content 33 ms, army content 83 ms, neutral content 33 ms and neutral content 83 ms). Motion parameters were included as covariates. We used predefined regions of interests (ROI) of the threat circuit that were previously found to be related to stress vulnerability and plasticity: the left amygdala (-28,0,-21), the left hippocampus (-25,-14,-21) and the vmPFC (0,50,-12)^19^. For each participant, beta weights were extracted and average,-d across all voxels within each ROI. Based on our previous findings that limbic activity changes due to stress exposure are only revealed in response to pictures from medical content that are presented closer to perceptual threshold (i.e., 33 ms)^19^, our analysis focused only on this condition and compared it to neutral stimuli presented for the same duration. Thus, each ROI's beta weights of the neutral 33 ms condition were subtracted from those of the medical 33 ms condition to reflect amygdala, hippocampus and vmPFC responses to emotional stimuli.

***Amygdala brain samples***

*RNA extraction*. RNA was extracted using the QIAGEN (Venlo, Netherlands) miRNeasy kit, ensuring full representation of all RNA length groups. Brain tissue was briefly homogenized with 700 µL QIAzol lysis buffer. Subsequently, homogenized tissue was lysed for 5 minutes at room temperature and mixed thoroughly with 140 µL of chloroform to fully allow neutralization. Following 3 min suspension, samples were centrifuged for 15 min (at 12,000× g and 4°C). The aqueous phase was next mixed with 1.5 volumes of 100% ethanol, loaded on a kit-provided RNA-binding spin column and briefly centrifuged for 30 sec at 8400× g. Following this, columns were washed by centrifugation for 30 sec at 8400xg, with 700 µL RWT buffer (containing 85% ethanol) and twice with 500 µL RPE buffer (containing 70% ethanol), in order to remove DNA and protein remnants, respectively. Flow-through was discarded after each centrifugation. Following these washing steps, further drying of the column was achieved by centrifuging it for 1 min at 21,067× g. RNA was eluted with 35 µL of nuclease-free water and was immediately put on ice to prevent degradation. RNA concentration was determined using Nanodrop-1000 (Thermo Scientific, Wlatham, MA, USA), and its integrity was assessed using an Agilent 2100 Bioanalyzer, which yielded RIN (RNA Integrity Number) values. RIN values were 5.2, ±1.4.

Prior to cDNA synthesis, DNA remnants were degraded by Sigma Aldrich (3,050 Spruce St., St. Louis, Missouri 63,103, United States) DNAse1 Amplification Grade (Sigma Aldrich). 500 ng RNA were diluted in 8 µL of double distilled, nuclease-free water, then mixed with 1 µL of 10x reaction buffer and 1 µL of DNAse (Sigma Aldrich). Following a 15 minutes incubation period at 25°c, addition of 1 µL of stop solution (Sigma Aldrich) terminated the DNAse reaction by incubation of 10 min at 70°c on a MJ Research PTC 200 Thermal Cycler (GMI Inc., 6511 Bunker Lake Blvd, Ramsey, MN 55303, United States). RNA concentration was measured again on the Nanodrop-1000 post DNAse treatment.

*hsa-miR-608 quantification using a Taqman qPCR assay*. cDNA was prepared from 10ng of RNA using Taqman MicroRNA reverse transcription kit (Applied Biosystems, CA, USA), in a volume of 10uL. cDNA synthesis reaction was performed on an MJ Research PTC 200 Thermal Cycler (GMI Inc., 6511 Bunker Lake Blvd, Ramsey, MN 55303, United States). Thermal cycler was programmed for 30 min at 16°c followed by 30 min at 42°c and finally 5 min at 85°c. For each technical repeat of the qPCR, 1.4 µL of cDNA product was mixed with 0.5 µL of x20 miR-608 Taqman primer, 3.1 µL DDW and 5 µL 2x PCR MasterMix. qPCR reactions were performed on a Biorad (Hercules, California, US) CFX96 Touch Real-Time PCR Detection System. Reaction began with enzyme heat activation at 95°c for 10 minutes, followed by 40 repeats of product amplification by 15 sec of 95°c followed by 60°c for 60 sec. Data was obtained using the Bio-Rad CFX Manager 3.0 software. Triplicate averages for all samples showed higher levels for human amygdala samples than for no reverse trascriptase or no template controls, indicating genuine miRNA-608 expression in all brain tissues tested.

*cDNA synthesis from brain RNA samples*. cDNA was prepared from 300 ng of RNA using the Quanta qScript cDNA Synthesis Kit (Quanta biosciences Inc., 202 Perry Parkway, Suite 1. Gaithersburg, MD 20877, USA). For each reaction 300 ng RNA were mixed with 4 µL ×5 Reaction Buffer, and 1 µL Reverse Transcriptase (except for the no-RT controls, where reverse transcriptase was not added), then double-distilled water were added for a final 20 µL reaction volume. Mixture was placed in a 200 µL PCR tube, and put in a MJ Research PTC 200 Thermal Cycler. Thermal Cycler was programmed for 5 min at 22°c followed by 30 min at 42°c and finally 5 min at 85°c. 180 µL DDW were then added to achieve 1:10 dilution.

*Quantitative real-time PCR and primers*. Quanta Perfecta SYBR green fastmix was used for both the targets and the beta-Actin gene used for normalization. 7.5 µL of SYBR supermix (Biorad Inc., Hercules, California, US), 0.75 µL of 10 µM of each left and right primers, and 6 µL of cDNA were used for each reaction. Reactions were performed on a Biorad CFX96 Touch Real-Time PCR Detection System. The same protocol was used for all targets. Starting by 3 minutes of heat activation at 95°c, 40 repeats of 95°c for 15 sec and 60°c for 30 sec were performed for product amplification, followed by melting curve measurements by gradually increasing the temperature from 67.0 to 94.6°c in 0.3°c increments performed every 5 sec. The data was then obtained using Bio-Rad CFX Manager 3.0 software. Triplicate PCR reactions were tested for each primer pair and tested samples. The primers (Sigma Aldrich (3,050 Spruce St., St. Louis, Missouri 63,103, United States)) used were as follows:

beta-Actin - Forward Primer - ; Reverse Primer -

AChE - Forward Primer - TCTCGAAACTACACGGCAGA; Reverse Primer - CGCAGGTCCAGACTAACGTA

CDC42 - Forward Primer - GCAGGGCAAGAGGATTATGA; Reverse Primer - CCCAACAAGCAAGAAAGGAG

NACC1 - Forward Primer - TGATGAACGTCGGCCAGAC; Reverse Primer -CGGATGCGATTTCGGGACT

TPP1 - Forward Primer - CCTCCACACGGTGCAAAAATG; Reverse Primer -CTCTGCTTGTCGGATGCTCAG

IL6 - Forward Primer - ACTCACCTCTTCAGAACGAATTG; Reverse Primer -CCATCTTTGGAAGGTTCAGGTTG

CD44 - Forward Primer - TGGTCGCTACAGCATCTCTC; Reverse Primer - CAGGTCTCAAATCCGATGCT

*Expression analysis*. Triplicates were averaged; sample averages of beta-Actin were subtracted from average target transcripts. Later, subtracted Cq means were normalized to zero by subtracting the value of one random sample from all samples. In order to create a linear expression value, 2 was raised to the power of the normalized cycle. Partial correlation of the expression levels, while accounting for RIN, was performed using SPSS. The effect was considered significant if p<0.05.

**Supplementary Table 1**

Clinical and RNA integrity data of brain-tissue volunteers, courtesy of the Netherland's Brain Bank

| **Autopsy Number (NBB)** | **Age** | **Gender** | **RNA Intergrity Number (RIN)** |
| --- | --- | --- | --- |
| S99/144 | 59 | F | 6.6 |
| S00/036 | 72 | F | 7.8 |
| S00/045 | 41 | F | 6.2 |
| S01/064 | 82 | M | 5.8 |
| S93/236 | 71 | F | 5.6 |
| S93/272 | 80 | M | 3 |
| S97/133 | 68 | M | 5.2 |
